# Supplementary material for: The landscape of antibody binding affinity in SARS-CoV-2 Omicron BA.1 evolution
Source: eLife. 2023 Feb 21;12:e83442. doi: 10.7554/eLife.83442 (PMC9949795; doi:10.7554/eLife.83442)
Supplement: Supplementary file 1. — The KD,app inferred from isogenic measurements (see Methods) shown with those inferred via Tite-seq measurement. NB denotes non-binding and SDs between replicates are also shown. [file elife-83442-supp1.docx]

| Strain | Antibody | Isogenic -log *K*_D,app_ | TiteSeq -log *K*_D,app_ |
| --- | --- | --- | --- |
| Omicron BA.1 | LY-CoV016 | NB | NB |
| Omicron BA.1 | LY-CoV555 | NB | NB |
| Omicron BA.1 | REGN10987 | NB | NB |
| Omicron BA.1 | S309 | 8.81 | 8.62 ± 0.18 |
| Wuhan Hu-1 | LY-CoV016 | 10.52 ± 0.24 | 10.27 ± 0.07 |
| Wuhan Hu-1 | LY-CoV555 | 10.01 ± 0.33 | 10.35 ± 0.02 |
| Wuhan Hu-1 | REGN10987 | 10.42 ± 0.49 | 10.52 ± 0.18 |
| Wuhan Hu-1 | S309 | 9.33 ± 0.27 | 9.00 ± 0.08 |
